# Supplementary material for: Predicting visual function by interpreting a neuronal wiring diagram
Source: Nature. 2024 Oct 2;634(8032):113–23. doi: 10.1038/s41586-024-07953-5 (PMC11446822; doi:10.1038/s41586-024-07953-5)
Supplement: Supplementary file 6 — CRF and ERF predictions for individual TmY4 and TmY9 cells. Analogous to Supplementary Data 3, but for TmY target types. Shown are the top four monosynaptic pathways, the strongest pathway passing through each of the top ten intermediary types (ranking from Extended Data Fig. 7), and the trisynaptic pathway Tm1–TmY–Dm3–TmY (see the section entitled Prediction of spatial normalization). [file 41586_2024_7953_MOESM6_ESM.zip › DataS4/TmY9q/720575940630961359.pdf]

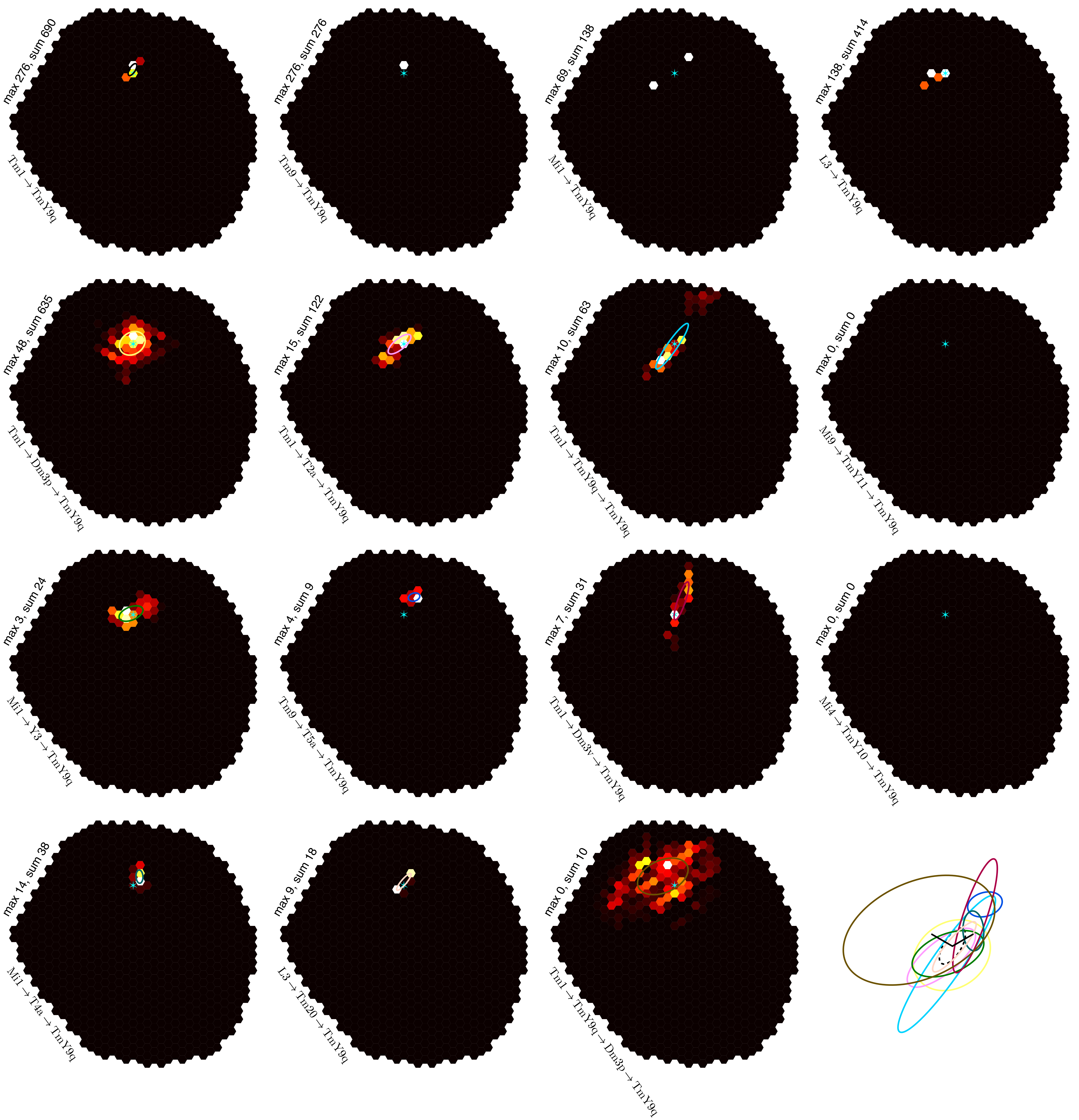

max 276, sum 690
Tm1 → TmY9q
max 276, sum 276
Tm9 → TmY9q
max 69, sum 138
Mi1 → TmY9q
max 138, sum 414
L3 → TmY9q
max 48, sum 635
Tm1 → Dm3p → TmY9q
max 15, sum 122
Tm1 → T2a → TmY9q
max 10, sum 63
Tm1 → TmY9q → TmY9q
max 0, sum 0
Mi9 → TmY11 → TmY9q
max 3, sum 24
Mi1 → Y3 → TmY9q
max 4, sum 9
Tm9 → T5a → TmY9q
max 7, sum 31
Tm1 → Dm3v → TmY9q
max 0, sum 0
Mi4 → TmY10 → TmY9q
max 14, sum 38
Mi1 → T4a → TmY9q
max 9, sum 18
L3 → Tm20 → TmY9q
max 0, sum 10
Tm1 → TmY9q → Dm3p → TmY9q
